# Inter-observer reproducibility of HER2 immunohistochemical assessment and concordance with fluorescent *in situ *hybridization (FISH): pathologist assessment compared to quantitative image analysis

**DOI:** 10.1186/1471-2407-9-165

**Published:** 2009-05-29

**Authors:** Gulisa Turashvili, Samuel Leung, Dmitry Turbin, Kelli Montgomery, Blake Gilks, Rob West, Melinda Carrier, David Huntsman, Samuel Aparicio

**Affiliations:** 1 Molecular Oncology and Breast Cancer Program, BC Cancer Research Centre, Vancouver, British Columbia, Canada; 2 Genetic Pathology Evaluation Centre, University of British Columbia, Vancouver, British Columbia, Canada; 3 Department of Pathology, Stanford University Medical Center, Stanford, California, USA

## Abstract

**Background:**

In breast cancer patients, HER2 overexpression is routinely assessed by immunohistochemistry (IHC) and equivocal cases are subject to fluorescent *in situ *hybridization (FISH). Our study compares HER2 scoring by histopathologists with automated quantitation of staining, and determines the concordance of IHC scores with FISH results.

**Methods:**

A tissue microarray was constructed from 1,212 invasive breast carcinoma cases with linked treatment and outcome information. IHC slides were semi-quantitatively scored by two independent pathologists on a range of 0 to 3+, and also analyzed with an Ariol automated system by two operators. 616 cases were scorable by both IHC and FISH.

**Results:**

Using data from unequivocal positive (3+) or negative (0, 1+) results, both visual and automated scores were highly consistent: there was excellent concordance between two pathologists (kappa = 1.000, 95% CI: 1-1), between two machines (kappa = 1.000, 95% CI: 1-1), and between both visual and both machine scores (kappa = 0.898, 95% CI: 0.775–0.979). Two pathologists successfully distinguished negative, positive and equivocal cases (kappa = 0.929, 95% CI: 0.909–0.946), with excellent agreement with machine 1 scores (kappa = 0.835, 95% CI: 0.806–0.862; kappa = 0.837, 95% CI: 0.81–0.862), and good agreement with machine 2 scores (kappa = 0.698, 95% CI: 0.6723–0.723; kappa = 0.709, 95% CI: 0.684–0.732), whereas the two machines showed good agreement (kappa = 0.806, 95% CI: 0.785–0.826). When comparing categorized IHC scores and FISH results, the agreement was excellent for visual 1 (kappa = 0.814, 95% CI: 0.768–0.856), good for visual 2 (kappa = 0.763, 95% CI: 0.712–0.81) and machine 1 (kappa = 0.665, 95% CI: 0.609–0.718), and moderate for machine 2 (kappa = 0.535, 95% CI: 0.485–0.584).

**Conclusion:**

A fully automated image analysis system run by an experienced operator can provide results consistent with visual HER2 scoring. Further development of such systems will likely improve the accuracy of detection and categorization of membranous staining, making this technique suitable for use in quality assurance programs and eventually in clinical practice.

## Background

HER2/neu (also known as c-erbB-2) is a member of the ErbB protein family, more commonly known as the epidermal growth factor receptor (EGFR) family. The HER2 protein is a cell membrane surface-bound receptor tyrosine kinase that is involved in signal transduction pathways leading to cell growth and differentiation [1]. HER2 is a proto-oncogene located on the long arm of human chromosome 17 (17q11.2-q12). Overexpression of the protein, typically caused by amplification of the HER2 gene, leads to constitutive activity of the HER2 receptor and breast tumor development through enhanced cell proliferation, survival, motility and adhesion [2]. HER2 gene amplification has been reported in 10–35% of invasive breast carcinomas, and it is associated with an aggressive disease course, increased disease recurrence, and decreased disease-free and overall survival in lymph node-positive patients [2-5]. In addition to its prognostic role, HER2 has now become more important as a predictive marker of treatment response to Trastuzumab, a humanized murine monoclonal antibody to the HER2 protein. In 1998, Trastuzumab (marketed as Herceptin, Genentech, San Francisco, California, USA) was approved for the targeted therapy of HER2-overexpressing metastatic breast cancer patients by the Food and Drug Administration (FDA) of the USA, and it has also recently been shown to be very effective in the adjuvant setting [2].

The effectiveness of Herceptin therapy depends on accurately evaluating HER2 status, which can be done either by immunohistochemical (IHC) assessment of HER2 protein expression or by evaluating HER2 gene amplification using *in situ *hybridization (ISH), most commonly, fluorescent ISH (FISH). FISH shows excellent sensitivity and specificity in detecting the HER2 gene amplification [6]. IHC assessment of HER2 status is an inexpensive and relatively standardized method that can be performed in all pathology laboratories. Of the various HER2 antibodies available, the FDA-approved Dako Herceptest (Dako, Glostrup, Denmark) has been considered the most reliable [7]. However, new antibodies such as Ventana PATHWAY anti-HER2/neu (4B5) rabbit monoclonal antibody also provide excellent sensitivity, specificity, and inter-laboratory reproducibility [8]. Based on the determination of staining intensity and percentage of cells with complete membrane staining, the results are scored semi-quantitatively on a range of 0 to 3+. According to these four-tier criteria, 0 and 1+ scores are considered negative, 3+ score is positive, while 2+ is equivocal (weakly positive) and requires confirmation by FISH [9-11]. The intraobserver reproducibility is generally satisfactory for both the percentage of positive cells and membrane staining [12-15]. The inter-observer agreement is excellent for scoring classes 0, 1+ and 3+ [11,16-19]. However, the determination of staining intensity and percentage of cells with complete membrane staining is subjective. This results in high inter-observer variability in assigning a 2+ score [11,17,20,21] and in discriminating between 2+ and 3+ classes [12]. Consequently this leads to a high rate of false-positives for intermediate IHC scores [22-24]. According to the HercepTest guidelines, cases with more than 10% of tumor cells showing strong circumferential membrane staining are classified as 3+. The American Society of Clinical Oncology/College of American Pathologists (ASCO/CAP) guidelines recommend using a 30% cut-off, in order to decrease the incidence of false positive cases [25].

It has been suggested that the use of digital microscopy improves the accuracy and inter-observer reproducibility of HER2 IHC analysis. Digital measurement of staining intensity is more accurate than assessment with a human eye because it is not influenced by factors such as the ambient light or pathologist fatigue [26,27]. We have recently shown that automated quantitation of estrogen receptor (ER) immunostaining yields results that do not differ from human scoring against dextran-coated charcoal biochemical assay and the most important clinico-pathologic correlate, patient outcome [28]. Consistent, objective and reproducible results for HER2 assessment can be generated by a number of available automated scoring systems such as the automated cellular imaging system (ACIS) (ChromaVision, Inc, San Juan Capistrano, California, USA) [29,30] optimized for use with Dako HercepTest, Micrometastasis Detection System (MDS, Applied Imaging, San Jose, California, USA) [31], Extended Slide Wizard (Tripath Imaging, Inc. Burlington, North Carolina, USA) and others [32-34].

To determine the inter-observer variability, we have compared results of visual and automated scoring of HER2 immunostaining on TMAs constructed from invasive breast carcinomas, with data from 1,413 cases used for FISH analysis. 616 cases were scorable by both methods. We then evaluated the concordance of IHC and FISH results and performed Kaplan-Meier survival analysis to determine the prognostic significance of different analyses of HER2 status.

## Methods

In this study, we used IHC data from 1,212 patients and FISH data from 616 patients. The data were derived from a series of 4,046 cases of invasive breast carcinoma diagnosed in 1986–1992, referred to the British Columbia Cancer Agency (BCCA) for treatment, and assembled into 17 tissue microarray (TMA) blocks. Ethical approval for the study was obtained from the Clinical Research Ethics Board of the BCCA [28]. Previously frozen breast cancer tissue samples were fixed in 10% neutral buffered formalin, embedded in paraffin and used to construct TMAs consisting of 0.6 mm tissue cores using a manual arrayer (Beecher Instruments, Inc., Silver Springs, Maryland, USA) as previously described [35,36].

From each TMA block, 4 μm thick sections were cut and immunostained on Ventana Benchmark XT staining system (Ventana Medical Systems, Tucson, Arizona, USA). Sections were deparaffinized in xylene, dehydrated through three alcohol changes and transferred to Ventana Wash solution. Endogenous peroxidase activity was blocked in 3% hydrogen peroxide. Slides were then incubated with Ventana PATHWAY anti-HER2/neu (4B5) rabbit monoclonal antibody at 37°C for 32 min and developed in DAB for 10 min. Finally, sections were counterstained with hematoxylin and mounted.

HER2 was scored visually by two independent pathologists (BG, GT) according to the HercepTest guidelines: 0 (negative): no staining is observed, or membrane staining is observed in <10% of the tumor cells; 1+ (negative): a faint/barely perceptible membrane staining is detected in >10% of tumor cells; the cells exhibit incomplete membrane staining; 2+ (weakly positive, equivocal): a weak to moderate complete membrane staining is observed in >10% of tumor cells; and 3+ (strongly positive): a strong complete membrane staining is observed in >10% of tumor cells. Only six 3+ cases (0.5%) showed heterogeneous staining, i.e. would have been interpreted as 2+ by ASCO/CAP guidelines. Therefore, the scoring system used in this study would not impact the results and conclusions. Scores were entered into a standardized Excel worksheet with a sector map matching each TMA section. Cases were not included in the statistical analysis if there was no tumor tissue in the cores or the cores were cut through. Original scoring grids were converted to tables using Deconvoluter 1.10 [37] and combined in a single text file with TMA-Combiner 1.00 [38]. The resulting text files were imported into SPSS 15.0 and R2.4.0 for Windows [39].

**Figure 1:**
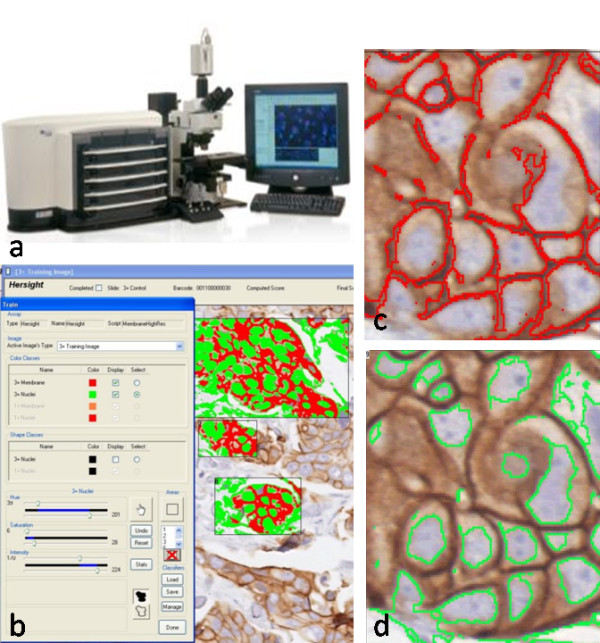
**Schematic illustration of automated HER2 scoring**. **a) **Image analysis system Ariol (Applied Imaging Inc., San-Jose, CA). **b) **Training window displaying the 3+ membrane and nuclear colors with fill mask. **c) **Outline of membrane as detected by the color classifier for the 3+ membrane color class. **d) **The border mask of nuclei as detected by the color classifier for the 3+ nuclei color class.

The same slides were digitized with a commercial image analysis system Ariol (Applied Imaging Inc., San-Jose, California, USA). For clinical lab applications, Ariol has received FDA clearance as an aid to pathologists in the detection, classification, and counting of cells of a particular color, intensity, size, pattern, and shape. Applied Imaging has received additional FDA 510(k) clearances for specific applications, including immunohistochemical assessment of HER2 in breast cancer. The Ariol system is based on an Olympus microscope with motorized stage and autofocus capabilities, and equipped with a black and white video camera. We regularly performed bright-field calibration using the Calibration slide to ensure accurate scanning and analysis. The system was set to Kohler illumination to capture high quality images. Slides were scanned at 20× objective magnification with three filters: red, green and blue. Ariol software, which converts these three-channel images into color reconstructions, was used for image analysis. The program was trained by a pathologist (DT) using representative cores containing areas that would be scored as 1+ and 3+ visually. Using the color pickup tool within the Ariol image analyzer, we selected membranes with weak positive staining and assigned "1+ intensity"; we then selected the membranes with strong positivity and assigned "3+ intensity". Similarly, we selected counterstained nuclei with the color pickup tool, and adjusted the desired size, roundness and other shape parameters under visual control. Numeric values for colors of the positive objects, i.e. membranes, and negative objects, i.e. nuclei, were stored on the hard drive in a color classifier file. Numeric values for the shape of the nuclei were stored in a separate shape classifier file. The program used these two files for segmentation of the nuclei and the membranes in all other cores, and these two files were sent out to be used in the machine 2. Scores from a "0" to a "3+" were automatically generated by the Ariol image analysis software for each core, based on the intensity and completeness of the positively stained membranes, and the percent of positive cells. The Ariol algorithm applies HercepTest criteria for the score calculations. Visual examples and a graphical explanation are given in Figure 1. The training step increases the specificity of the analysis as it ensures that extracellular matrix and most stromal cells are excluded from image analysis, and it allows the program to calculate percent of positive tumor cells more precisely. After the program training on one of the representative TMA cores, the rest of the analysis was performed without human supervision. All tissue cores were analyzed in toto; no specific pathologist selection of tumor tissue within the cores was made following the training step. For statistical analysis, we selected only cores with at least 50 tumor cells detected, i.e. all cores with less than 50 cells were considered unscorable. To get an estimate of the demands posed on the operator of the Ariol system, the same slides were scanned and processed on an identical Ariol system by an operator with less than one week experience working with this particular Ariol script (KM). The descriptors of the color and shape of the positive and negative tumor cells were transferred from one system to another, therefore variations in the image analysis results depended only on the scanner settings, i.e. brightfield calibration, positioning and white balance, but not on the image analysis settings.

The hematoxylin and eosin and IHC images of all cores used in this study are publicly available at the companion site [40]. The site was constructed using Genetic Pathology Evaluation Centre (GPEC) database and a Java applet provided by Bacus Laboratories, Inc. All slides were scanned with a BLISS scanner (Bacus Laboratories, Inc., Lombard, Illinois, USA), and posted on the site. WebSlide Browser for Windows (Bacus Laboratories, Inc., Lombard, Illinois, USA) can be used for viewing preview images of the arrays and images of individual cores.

Six-micron sections of the TMA slides were hybridized with probes to LSI HER2 and CEP17 with the PathVysion™ HER2 DNA Probe Kit using a modified protocol, as previously described [41]. Analysis of FISH signals was performed using Metasystems™ automated image acquisition and analysis system, Metafer (Metasystems, Altlussheim, Germany). This automated system scores FISH signals by employing specific measurement algorithms to detect and quantify clustered signals. Average copy number for each probe was calculated and the amplification ratio (ratio between the average copy per cell for Her2 and the average copy for centromere 17) determined (MC). HER2 amplification was defined as a HER2/CEP17 ratio of 2.2 or more. A HER2/CEP17 ratio <1.8 was considered negative for HER2 amplification, and a ratio at or near the cut-off (1.8–2.2) was interpreted as equivocal. Tumors that failed to hybridize were not included in the analysis. We only accepted scores if >40 tiles were counted. With Metafer system, one tile is considered one cell as the size of a tile is approximately the average size of a nucleus. Normal cells were excluded wherever possible, and the corresponding H&E slides were reviewed when needed.

For statistical analysis, we used data from 1212 patients for the IHC and 616 patients for the IHC/FISH comparisons. Exclusion criteria included core drop-off during processing, insufficient or absent tumor tissue within the cores, and artifactual distortion of the tissue making discrimination of cellular structure impossible. Statistical analysis was performed in SPSS 15.0 for Windows (SPSS Inc., Chicago, Illinois) and R 2.4.0 [39]. All tests were two-sided and used a 5% alpha level to determine significance. 95% bootstrapped confidence intervals were calculated using the adjusted bootstrap percentile (bias-corrected and accelerated) method [42]. Breast cancer specific survival was estimated using Kaplan-Meier curves and survival differences were determined by log-rank tests. We used the open-source R 2.4.0 package to calculate differences between kappa statistics from visual to automated scoring comparisons; a permutation test with 10,000 permutations was implemented.

## Results

### IHC and FISH results

The number of cases scorable by all four observers (visual or machine) on IHC slides, regardless of FISH status was 1,212 (30%). Of 4,046 cases analyzed, FISH was successfully performed in 1413 cases (34.9%). Of 1,413 FISH scorable cases, HER2 was amplified (HER2/CEP17 ratio of 2.2 or more) in 252 cases (17.8%). Borderline HER2 amplification (HER2/CEP17 ratio 1.8–2.2) was seen in 77 cases (5.4%), and 1084 cases (76.7%) were found to be non-amplified (HER2/CEP17 ratio <1.8). The number of cases scorable by both IHC and FISH, including FISH equivocal cases, was 616. Table 1 shows the full breakdown of data by FISH and IHC scored by the four observers.

**Table 1:** Comparison of FISH and IHC results in 616 cases

FISH-amplified (n = 185)				
	0	1+	2+	3+

Visual 1	9 (4.9%)	9 (4.9%)	30 (16.2%)	137 (74.1%)

Visual 2	19 (10.3%)	12 (6.5%)	29 (15.7%)	125 (67.6%)

Machine 1*	0 (.0%)	36 (19.5%)	79 (42.7%)	70 (37.8%)

Machine 2*	0 (.0%)	41 (22.2%)	137 (74.1%)	7 (3.8%)

**FISH-non-amplified (n = 394)**				

	0	1+	2+	3+

Visual 1	293 (74.4%)	78 (19.8%)	15 (3.8%)	8 (2.0%)

Visual 2	315 (79.9%)	59 (15.0%)	13 (3.3%)	7 (1.8%)

Machine 1*	1 (0.3%)	372 (94.4%)	17 (4.3%)	4 (1.0%)

Machine 2*	4 (1.0%)	375 (95.2%)	15 (3.8%)	0 (.0%)

**FISH-equivocal (n = 37)**				

	0	1+	2+	3+

Visual 1	14 (37.8%)	12 (32.4%)	7 (18.9%)	4 (10.8%)

Visual 2	18 (48.6%)	8 (21.6%)	6 (16.2%)	5 (13.5%)

Machine 1*	0 (.0%)	25 (67.6%)	9 (24.3%)	3 (8.1%)

Machine 2*	2 (5.4%)	23 (62.2%)	11 (29.7%)	1 (2.7%)

*Only cores with more than 50 cells were considered scorable on the Ariol system.

### Analysis of HER2 IHC inter-observer variability by Kappa statistics

Inter-observer variability was estimated by comparing the visual scores of two pathologists, and the automated scores generated by two operators on two different Ariol hardware systems. Comparison of categorized variables ({0, 1+} versus {2+} versus {3+}) from 1,212 patients using weighted kappa statistics (R function wkappa(ψ) using squared weights) showed excellent inter-observer agreement: for visual 1 versus visual 2 scores, kappa = 0.929 (95% CI: 0.909–0.946), visual 1 versus machine 1 scores, kappa = 0.835 (95% CI: 0.806–0.862), and visual 2 versus machine 1 scores, kappa = 0.837 (95% CI: 0.81–0.862); good agreement was seen between machine 2 and visual 1, kappa = 0.698 (95% CI: 0.672–0.723), machine 2 and visual 2, kappa = 0.709 (95% CI: 0.684–0.732), and machine 1 and machine 2, kappa = 0.806 (95% CI: 0.785–0.826) (Table 2).

**Table 2:** Weighted Kappa statistics on the whole cohort for comparison of inter-observer concordance for categorized HER2 IHC variables (n = 1212)

	Visual 1	Visual 2	Machine 1
**Visual 1**	-	-	-
**Visual 2**	0.929 (0.909 – 0.946)	-	-
**Machine 1**	0.835 (0.806 – 0.862)	0.837 (0.810 – 0.862)	-
**Machine 2**	0.698 (0.672 – 0.723)	0.709 (0.684 – 0.732)	0.806 (0.785 – 0.826)

When comparing binarized IHC scores (0, 1+ {negative} versus 3+ {positive}) in a set of 849 patients (363 cases with 2+ scores were excluded), the kappa values were within 'excellent' agreement range: for two visual scores, kappa = 1.000 (95% CI: 1-1); for two machine scores, kappa = 1.000 (95% CI: 1-1); for visual 1 versus both machine scores, kappa = 0.898 (95% CI: 0.775–0.979); and for visual 2 versus both machine scores, kappa = 0.898 (95% CI: 0.775–0.979), (Table 3).

**Table 3:** Kappa statistics for comparison of inter-observer concordance for binarized HER2 IHC variables (n = 849)

	Visual 1	Visual 2	Machine 1
**Visual 1**	-	-	-
**Visual 2**	1.000 (1 - 1)	-	-
**Machine 1**	0.898 (0.775 – 0.979)	0.898 (0.775 – 0.979)	-
**Machine 2**	0.898 (0.775 – 0.979)	0.898 (0.775 – 0.979)	1.000 (1 - 1)

We also performed Kappa permutation test to assess whether the HER2 IHC scores differed in their ability to match the gold standard. This test included categorized variables (n = 352) to assess the ability of the HER2 score to indicate negative (0, 1+) versus equivocal (2+) versus positive (3+) cases where visual 1 IHC score is the gold standard (Table 4). The permutation test could not be done for binarized IHC scores because there were only 229 cases available for analysis when visual 1 IHC was used as the gold standard, and 382 cases were available when FISH was used as the gold standard. There were no discrepant cases between visual 1 and visual 2, with only one discrepant case between both visual scores and both machines.

**Table 4:** Permutation test to determine the inter-observer variability for categorized IHC variables (n = 352)

	Visual 1	Visual 2	Machine 1
**Visual 1**	-	-	-
**Visual 2**	0.426	-	-
**Machine 1**	1 × 10^-4^	0.001	-
**Machine 2**	1 × 10^-4^	1 × 10^-4^	1 × 10^-4^

### Concordance of IHC and FISH results by Kappa statistics

The concordance of IHC and FISH results was analyzed using binarized and categorized variables by Kappa statistics. When comparing categorized IHC scores (0, 1+ (negative) versus 2 (equivocal) versus 3+ (positive)) with FISH results in a set of 616 patients, the agreement was excellent for visual 1 (kappa = 0.814, 95% CI: 0.768–0.856), good for visual 2 (kappa = 0.763, 95% CI: 0.712–0.81), and machine 1 (kappa = 0.665, 95% CI: 0.609–0.718), while machine 2 showed moderate agreement with FISH results (kappa = 0.535, 95% CI: 0.485–0.584) (Table 5).

**Table 5:** Concordance of IHC and FISH results by Kappa statistics

	FISH vs categorized IHC (n = 616)	FISH vs binarized IHC (n = 382)
**Visual 1**	0.814 (0.768 – 0.856)	0.328 (0.0955 – 0.537)
**Visual 2**	0.763 (0.712 – 0.81)	0.328 (0.0914 – 0.538)
**Machine 1**	0.665 (0.609 – 0.718)	0.343 (0.101 – 0.558)
**Machine 2**	0.535 (0.485 – 0.584)	0.343 (0.0935 – 0.555)

When comparing binarized IHC scores (0, 1+ {negative} versus 3+ {positive}) and FISH results in a set of 382 patients (234 cases with 2+ scores were excluded), FISH data only showed fair agreement with all four IHC scores: visual 1 (kappa = 0.328, CI: 0.0955 – 0.537), visual 2 (kappa = 0.328, CI: 0.0914 – 0.538), machine 1 (kappa = 0.343 (0.101 – 0.558), and machine 2 (kappa = 0.343 (0.0935 – 0.555) (Table 5). This was likely caused by the large number of 2+ scores excluded (n = 234) and low number of 3+ scores (n = 6) available for this analysis. Therefore, the proportion of HER2-positive and HER2-negative cases was not fairly represented for the concordance analysis of the binarized data.

The clinical consequences of using a machine for HER2 scoring are summarized in Table 6. Automated scoring on the Ariol machine would result in more 2+ scores (2–3 times as many as visual scoring) with a consequent increase of FISH assessments in clinical practice.

**Table 6:** Comparison of automated IHC scores with visual scores and FISH results

IHC/FISH	Machine 1	Machine 2	Visual 1	Visual 2	FISH
**Negative (IHC 0,1+/FISH <1.8)**	434	445	415	431	394

**Equivocal (IHC 2+/FISH 1.8–2.2)**	105	163	52	48	37

**Positive (IHC 3+/FISH >2.2)**	77	8	149	137	185

### Kaplan-Meier survival analysis

For 1,212 patients whose tissue cores were scorable by all four observers on IHC slides, median age at diagnosis was 59 years, and median follow-up time was 12.24 years. Clinical-pathological characteristics of these patients are summarized in Table 7.

**Table 7:** Clinical-pathological characteristics of 1212 patients

Variables		**No**.	%
Menstrual status	Premenopausal	368	30.4
	Postmenopausal	811	66.9
	Unknown	33	2.7

LN status	Negative	645	53.2
	Positive	563	46.5
	Unknown	4	0.3

Histological diagnosis	Ductal	1149	94.8
	Lobular	48	4
	Other	15	1.2

Systemic therapy	No adjuvant systemic therapy	484	39.9
	Tamoxifen, no chemotherapy	398	32.8
	Chemotherapy, no Tamoxifen	228	18.8
	Chemotherapy and Tamoxifen	100	8.3
	Ovarian ablation or hormono-therapy other than Tamoxifen, no chemotherapy	1	0.1
	Ovarian ablation or hormono-therapy other than Tamoxifen, and chemotherapy	1	0.1

**Figure 2:**
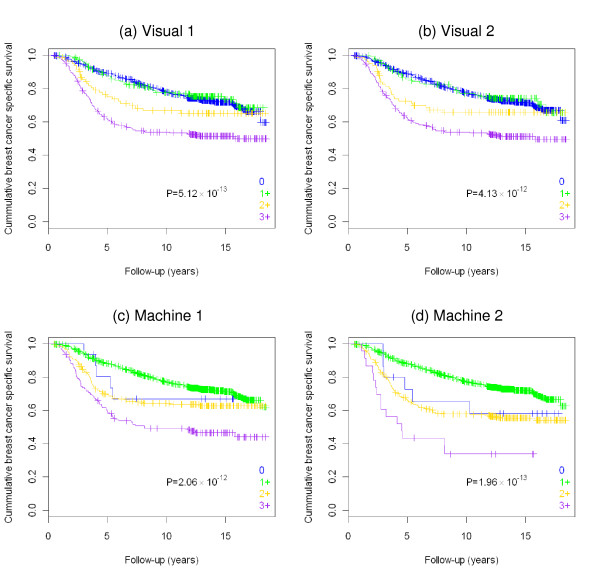
**Kaplan-Meier survival analysis performed on the data categorized as negative (0, 1+), equivocal (2+) and positive (3+) (n = 1210)**. **a) **Visual scoring #1. **b) **Visual scoring #2. **c) **Automated system #1. **d) **Automated system #2.

**Figure 3:**
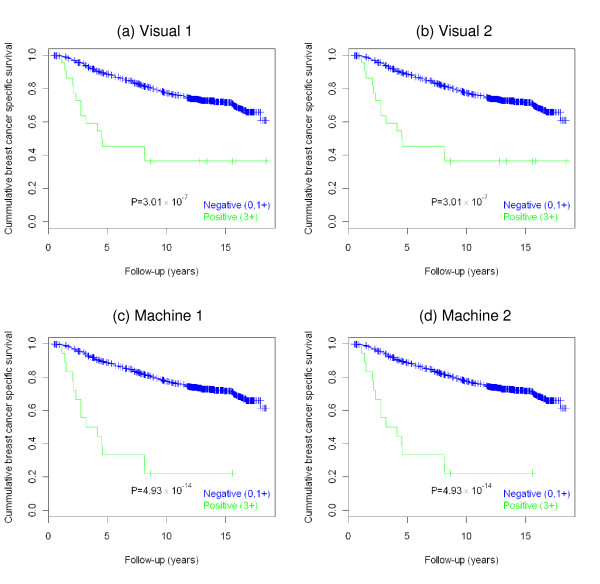
**Kaplan-Meier survival analysis performed on the binarized data (negative {0, 1+} and positive {3+}) (n = 848)**. **a) **Visual scoring #1. **b) **Visual scoring #2. **c) **Automated system #1. **d) **Automated system #2.

Kaplan-Meier survival analysis of cases stratified based on the HER2 status, as determined by visual or machine scoring of the immunostained slides, is shown in Figure 2. Results of the log-rank tests with P values in a set of 1,210 patients (outcome information was not available in 2 cases), stratified as 0 (negative), 1+ (weak), 2+ (equivocal) and 3+ (positive) are as follows: visual scoring 1 χ^2 ^= 60.281, P = 5.12 × 10^-13^; visual scoring 2 χ^2 ^= 56.037, P = 4.13 × 10^-12^; machine scoring 1 χ^2 ^= 57.453, P = 2.06 × 10^-12^; machine scoring 2 χ^2 ^= 62.232, P = 1.96 × 10^-13 ^(Figure 2). After binarization of the scores as either HER2-positive or HER2-negative in a set of 848 patients, the results of log-rank test were: visual scoring 1 χ^2 ^= 26.245, P = 3.01 × 10^-7^; visual scoring 2 χ^2 ^= 26.245, P = 3.01 × 10^-7^; machine scoring 1 χ^2 ^= 56.757, P = 4.93 × 10^-14^; machine scoring 2 χ^2 ^= 56.757, P = 4.93 × 10^-14 ^(Figure 3).

The permutation analysis in a set of 615 patients (outcome information was not available for one patient) showed that the differences in prognostic significance of these different analyses of HER2 status are not statistically significant, i.e. visual and machine scoring show similar results for categorized variables (Table 8). The permutation analysis could not be performed for binarized variables because after excluding 2+ scores, only 382 cases were available for analysis and there were no discrepant cases between the visual scores and between the machine scores, only 1 discrepant score between visual 1 and machine 1, and 19 discrepent scores between visual 1 and FISH results.

**Table 8:** Permutation test to compare the differences between categorized IHC and FISH results using survival outcome as the gold standard (n = 615)

	FISH	Visual 1	Visual 2	Machine 1
**FISH**	-	-	-	-
**Visual 1**	0.352	-	-	-
**Visual 2**	0.562	0.695	-	-
**Machine 1**	0.441	0.982	0.811	-
**Machine 2**	0.214	0.472	0.332	0.484

## Discussion

In breast cancer patients, determination of prognosis and treatment strategies based on HER2 status greatly depends on the accurate evaluation of HER2 overexpression by IHC and/or FISH. HER2 immunohistochemistry is an inexpensive method that can be performed readily in all pathology laboratories on either standard paraffin sections or TMA sections [43]. However, consensus regarding the best methods, reagents, or cut-off points to determine HER2 status is still debated [25,28,44-46]. TMAs are useful for the assessment of automated unsupervised image analysis systems because of the careful selection of the areas of interest, the identical staining conditions for all cores on a single slide, and the small size of the tissue cores representable by a single image [37,38,47]. Problems inherent in TMA studies include taking cores from the non-cancerous areas, and a loss of cores during the staining procedure. We analyzed the results of visual (two pathologists) and automated (two operators on the Ariol image analysis system) scoring of HER2 immunostaining. Since only cores with more than 50 tumor cells detected were considered scorable on the Ariol system, the number of cases scorable by all four observers was 1,212. FISH was successfully performed in 1,413 cases (34.9%) with an amplification rate of 17.8%, which is within the reported range of 10–35% [2-5].

When using the four-tier criteria for HER2 IHC (0 and 1+ negative, 3+ positive, and 2+ equivocal), the inter-observer agreement is usually excellent for negative (0, 1+) and positive (3+) cases [11,16-19]. To estimate the inter-observer variability in our study, we analyzed the results of two visual and two automated scores. When comparing binarized IHC scores, the inter-observer agreement was excellent between the two pathologists (kappa = 1.000, 95% CI: 1-1), between the two machines (kappa = 1.000, 95% CI: 1-1), between both visual and both machine scores (kappa = 0.898, 95% CI: 0.775–0.979). This suggests that the Ariol automated system can be used successfully for scoring clearly positive or negative cases, whereas equivocal cases will always need follow-up through pathologist review and/or FISH.

Since the evaluation of staining intensity and percentage of cells with complete membrane positivity is subjective, the inter-observer variability tends to be higher for scoring 2+ cases [11,17,20,21] and discriminating 1+ and 2+ [48] or 2+ and 3+ cases [12]. The percentage of disagreement in intraobserver reproducibility ranges from 0.9% to 3.7%. It is recommended that two expert pathologists evaluate all slides with a double-blind method and discuss discordant cases [49]. In our study, the inter-observer agreement was excellent for categorized variables (0, 1+ versus 2+ versus 3+) between the two pathologists (kappa = 0.929, 95% CI: 0.909–0.946). The first machine scores also showed excellent agreement with both pathologists (kappa = 0.835, 95% CI: 0.806–0.862; kappa = 0.837, 95% CI: 0.81–0.862). The worst concordance for categorized variables was observed between the second machine operated by a less experienced operator and either pathologist 1 (kappa = 0.698, 95% CI: 0.6723–0.723) or pathologist 2 (kappa = 0.709, 95% CI: 0.684–0.732) or the first machine scores (kappa = 0.806, 95% CI: 0.785–0.826). Although these kappa values are still considered to be in good agreement, it is likely that lack of experience in operating the Ariol system using particular scripts can influence the results of automated scoring for categorized variables. However, the results of the IHC analysis for categorized scores by either pathologists or machines demonstrated similar accuracy in assessment of prognostic significance of HER2 expression in Kaplan-Meier analysis.

Discrepancies between HER2 IHC and FISH results are not uncommon and may be caused by errors in manual IHC interpretation, IHC reagent limitations [50,51], different anti-HER2 primary antibodies [48,52-57], a lack of interlaboratory standardization of IHC and reproducibility in interpretation of the results [58,59]. When comparing categorized IHC scores and FISH results, only pathologist 1 showed excellent agreement with FISH results (kappa = 0.814, 95% CI: 0.768–0.856). There was good agreement between FISH and pathologist 2 scores (kappa = 0.763, 95% CI: 0.712–0.81), and machine 1 scores (kappa = 0.665, 95% CI: 0.609–0.718), while the less experienced operator showed moderate agreement with FISH results (kappa = 0.535, 95% CI: 0.485–0.584). In addition to the amount of experience working with particular Ariol scripts, variations in the image analysis results may depend on the scanner settings, such as calibration, positioning and white balance because the image analysis settings were transferred from the first Ariol system to the other, without training the program. It should also be noted that HER2 gene amplification is not always accompanied by protein overexpression and vice versa. The poor prognosis associated with HER2 amplification may be attributed to global genomic instability, as cells with high frequencies of chromosomal alterations are associated with increased cellular proliferation and aggressive behavior. This suggests that HER2 amplification may serve as a surrogate marker for underlying genomic instability [60]. The discrepancy between FISH and IHC results can also be explained by technical and interpretational limitations such as failure to hybridize, scoring algorithm on the Metafer system, small size of the TMA core making this small region not representative for the tumor. For categorized variables, comparison of log-rank tests with 10,000 permutations detected no significant differences among four observers. Two pathologists successfully distinguished negative, positive and equivocal cases, but automated scoring led to 2–3 times as many 2+ cases as visual scoring. This suggests that fully automated scoring, regardless of use experience, does not provide better distinction of 2+ cases in our study. This is inconsistent with previously reported results that machine scoring of HER2 is reproducible for 2+ cases [61]. However, the latter study only analyzed 65 cases using an Extended Slide Wizard (Tripath Imaging, Inc., Burlington, North Carolina, USA) workstation running prototype software. In theory, computer-assisted image analysis should provide more accurate results for IHC quantitation, in comparison with semiquantitative scoring by a pathologist, as image analysis systems can measure the intensity of staining much more precisely than a human eye [62]. In practice, however, the accuracy of automated quantitative analysis depends on a variety of factors other than technical issues. Fully automated systems cannot distinguish between malignant and benign lesions with a precision comparable to the expertise level of a pathologist [63,64], and require pathologist input to identify the area to be analyzed. Since the machine interprets most visual 3+ scores as 2+, it is likely that automated HER2 scoring on the Ariol system would result in more FISH assessments in clinical practice. The automated system also leads to more 1+ cases in comparison to visual scoring, which may give rise to more FISH-amplified cases to be scored as 1+ (negative). However, this would not change patient management for 0 and 1+ cases as these are both interpreted as negative.

## Conclusion

The present study shows that fully automated image analysis with a system operated by an experienced operator, but without continuous human supervision, can provide results consistent with the scoring of HER2 immunostaining by pathologists. The inter-observer agreement was excellent between the two pathologists and between the experienced operator and the pathologists for both binarized and categorized HER2 scores, as well as between the two machines for binarized scores. There was a good agreement between the two machines, and between the less experienced operator and the pathologists for categorized HER2 scores. We have previously reported that automated quantitation of ER immunostaining on the same TMA series can produce results that do not differ from pathologist scoring and dextran-coated charcoal biochemical assay [28]. Unlike ER quantitation, automated scoring of HER2 staining on the Ariol system did not provide excellent agreement between machine scores or the gold standard FISH. Although Kaplan-Meier analysis showed similar accuracy of visual and machine scores in assessment of prognostic significance of HER2 status for categorized IHC variables, the automated quantitation could not distinguish 2+ scores better than the pathologists. It resulted in more 2+ cases which would lead to more FISH assessments in clinical practice. Further development of image analysis systems will likely improve the accuracy of detection and categorization of membranous staining in histological sections, making this technique more sensitive, specific and thus suitable for use in quality assurance programs.

## List of abbreviations

ASCO/CAP: American society of clinical oncology/college of American pathologists; ACIS: automated cellular imaging system; BCCA: British Columbia Cancer Agency; EGFR: epidermal growth factor receptor; ER: estrogen receptor; FISH: fluorescent *in situ *hybridization; FDA: food and drug administration; GPEC: genetic pathology evaluation centre; IHC: immunohistochemistry; ISH: *in situ *hybridization; MDS: Micrometastasis Detection System; TMA: tissue microarray.

## Competing interests

The authors declare that they have no competing interests.

## Authors' contributions

GT drafted the manuscript and visually scored the sections. SL performed statistical analysis of the data and assisted in drafting the manuscript. DT and KM operated automated image analysis system. BG visually scored the sections, participated in study design and coordination, and assisted in drafting manuscript. RW helped to score the sections. MM performed FISH analysis. DH and SA conceived of the study, participated in its design and coordination, and assisted in editing the manuscript. All authors read and approved the final manuscript.

## Pre-publication history

The pre-publication history for this paper can be accessed here:

http://www.biomedcentral.com/1471-2407/9/165/prepub

## References

[B1] Olayioye MA Update on HER-2 as a target for cancer therapy: intracellular signaling pathways of ErbB2/HER-2 and family members Breast Cancer Res 2001 3 6 385 389 11737890 10.1186/bcr327 PMC138705

[B2] Laudadio J Quigley DI Tubbs R Wolff DJ HER2 testing: a review of detection methodologies and their clinical performance Expert Rev Mol Diagn 2007 7 1 53 64 10.1586/14737159.7.1.53 17187484

[B3] Ross JS Fletcher JA Linette GP Stec J Clark E Ayers M Symmans WF Pusztai L Bloom KJ The Her-2/neu gene and protein in breast cancer 2003: biomarker and target of therapy Oncologist 2003 8 4 307 325 10.1634/theoncologist.8-4-307 12897328

[B4] Zhou BP Hung MC Dysregulation of cellular signaling by HER2/neu in breast cancer Semin Oncol 2003 30 5 Suppl 16 38 48 10.1053/j.seminoncol.2003.08.006 14613025

[B5] Menard S Casalini P Campiglio M Pupa SM Tagliabue E Role of HER2/neu in tumor progression and therapy Cell Mol Life Sci 2004 61 23 2965 2978 10.1007/s00018-004-4277-7 15583858 PMC11924417

[B6] Downs-Kelly E Pettay J Hicks D Skacel M Yoder B Rybicki L Myles J Sreenan J Roche P Powell R Analytical validation and interobserver reproducibility of EnzMet GenePro: a second-generation bright-field metallography assay for concomitant detection of HER2 gene status and protein expression in invasive carcinoma of the breast Am J Surg Pathol 2005 29 11 1505 1511 10.1097/01.pas.0000172294.67409.4f 16224218

[B7] Rhodes A Jasani B Anderson E Dodson AR Balaton AJ Evaluation of HER-2/neu immunohistochemical assay sensitivity and scoring on formalin-fixed and paraffin-processed cell lines and breast tumors: a comparative study involving results from laboratories in 21 countries Am J Clin Pathol 2002 118 3 408 417 10.1309/97WN-W6UX-XJWT-02H2 12219783

[B8] Powell WC Hicks DG Prescott N Tarr SM Laniauskas S Williams T Short S Pettay J Nagle RB Dabbs DJ A new rabbit monoclonal antibody (4B5) for the immunohistochemical (IHC) determination of the HER2 status in breast cancer: comparison with CB11, fluorescence in situ hybridization (FISH), and interlaboratory reproducibility Appl Immunohistochem Mol Morphol 2007 15 1 94 102 10.1097/PAI.0b013e31802ced25 17536315

[B9] Garcia-Caballero T Menendez MD Vazquez-Boquete A Gallego R Forteza J Fraga M HER-2 status determination in breast carcinomas. A practical approach Histol Histopathol 2006 21 3 227 236 16372244 10.14670/HH-21.227

[B10] Penault-Llorca F Balaton A Sabourin JC Le Doussal V [Immunochemistry evaluation of HER2 status in infiltration breast cancer: technical protocol and interpretation guidelines] Ann Pathol 2002 22 2 150 157 12124503

[B11] McCormick SR Lillemoe TJ Beneke J Schrauth J Reinartz J HER2 assessment by immunohistochemical analysis and fluorescence in situ hybridization: comparison of HercepTest and PathVysion commercial assays Am J Clin Pathol 2002 117 6 935 943 10.1309/3643-F955-7Q6B-EWWL 12047146

[B12] Interobserver reproducibility of immunohistochemical HER-2/neu assessment in human breast cancer: an update from INQAT round III Int J Biol Markers 2005 20 3 189 194 28207128 10.5301/JBM.2008.4178

[B13] Interobserver reproducibility of immunohistochemical HER-2/neu evaluation in human breast cancer: the real-world experience Int J Biol Markers 2004 19 2 147 154 15255548 10.1177/172460080401900210

[B14] Kay EW Walsh CJ Cassidy M Curran B Leader M C-erbB-2 immunostaining: problems with interpretation J Clin Pathol 1994 47 9 816 822 7962650 10.1136/jcp.47.9.816 PMC494938

[B15] Nichols DW Wolff DJ Self S Metcalf JS Jacobs D Kneuper-Hall R Cate JCt A testing algorithm for determination of HER2 status in patients with breast cancer Ann Clin Lab Sci 2002 32 1 3 11 11848614

[B16] Hsu CY Ho DM Yang CF Lai CR Yu IT Chiang H Interobserver reproducibility of Her-2/neu protein overexpression in invasive breast carcinoma using the DAKO HercepTest Am J Clin Pathol 2002 118 5 693 698 10.1309/6ANB-QXCF-EHKC-7UC7 12428788

[B17] Lacroix-Triki M Mathoulin-Pelissier S Ghnassia JP Macgrogan G Vincent-Salomon A Brouste V Mathieu MC Roger P Bibeau F Jacquemier J High inter-observer agreement in immunohistochemical evaluation of HER-2/neu expression in breast cancer: a multicentre GEFPICS study Eur J Cancer 2006 42 17 2946 2953 10.1016/j.ejca.2006.06.020 16989997

[B18] Tsuda H Akiyama F Terasaki H Hasegawa T Kurosumi M Shimadzu M Yamamori S Sakamoto G Detection of HER-2/neu (c-erb B-2) DNA amplification in primary breast carcinoma. Interobserver reproducibility and correlation with immunohistochemical HER-2 overexpression Cancer 2001 92 12 2965 2974 11753973 10.1002/1097-0142(20011215)92:12<2965::AID-CNCR10156>3.0.CO;2-A

[B19] Rodriguez Moguel L Vega Ramos B [Reproducibility of Her-2/neu overexpression with HERCEP test in invasive ductal breast cancer] Ginecol Obstet Mex 2002 70 601 606 12661333

[B20] Dolan M Snover D Comparison of immunohistochemical and fluorescence in situ hybridization assessment of HER-2 status in routine practice Am J Clin Pathol 2005 123 5 766 770 10.1309/Q0DGL26RUCK1K5EV 15981817

[B21] Diaz NM Laboratory testing for HER2/neu in breast carcinoma: an evolving strategy to predict response to targeted therapy Cancer Control 2001 8 5 415 418 11579337 10.1177/107327480100800504

[B22] Jacobs TW Gown AM Yaziji H Barnes MJ Schnitt SJ Specificity of HercepTest in determining HER-2/neu status of breast cancers using the United States Food and Drug Administration-approved scoring system J Clin Oncol 1999 17 7 1983 1987 10561248 10.1200/JCO.1999.17.7.1983

[B23] Leong AS Formby M Haffajee Z Clarke M Morey A Refinement of immunohistologic parameters for Her2/neu scoring validation by FISH and CISH Appl Immunohistochem Mol Morphol 2006 14 4 384 389 10.1097/01.pai.0000210415.53493.d4 17122633

[B24] Tubbs RR Pettay JD Roche PC Stoler MH Jenkins RB Grogan TM Discrepancies in clinical laboratory testing of eligibility for trastuzumab therapy: apparent immunohistochemical false-positives do not get the message J Clin Oncol 2001 19 10 2714 2721 11352964 10.1200/JCO.2001.19.10.2714

[B25] Wolff AC Hammond ME Schwartz JN Hagerty KL Allred DC Cote RJ Dowsett M Fitzgibbons PL Hanna WM Langer A American Society of Clinical Oncology/College of American Pathologists guideline recommendations for human epidermal growth factor receptor 2 testing in breast cancer J Clin Oncol 2007 25 1 118 145 10.1200/JCO.2006.09.2775 17159189

[B26] Rinner O Gegenfurtner KR Time course of chromatic adaptation for color appearance and discrimination Vision Res 2000 40 14 1813 1826 10.1016/S0042-6989(00)00050-X 10837828

[B27] Byrne A Hilbert DR Color realism and color science Behav Brain Sci 2003 26 1 3 21 14598439 10.1017/s0140525x03000013

[B28] Turbin DA Leung S Cheang MC Kennecke HA Montgomery KD McKinney S Treaba DO Boyd N Goldstein LC Badve S Automated quantitative analysis of estrogen receptor expression in breast carcinoma does not differ from expert pathologist scoring: a tissue microarray study of 3,484 cases Breast Cancer Res Treat 2008 110 3 417 26 10.1007/s10549-007-9736-z 17912629

[B29] Ciampa A Xu B Ayata G Baiyee D Wallace J Wertheimer M Edmiston K Khan A HER-2 status in breast cancer: correlation of gene amplification by FISH with immunohistochemistry expression using advanced cellular imaging system Appl Immunohistochem Mol Morphol 2006 14 2 132 137 10.1097/01.pai.0000150516.75567.13 16785779

[B30] Tawfik OW Kimler BF Davis M Donahue JK Persons DL Fan F Hagemeister S Thomas P Connor C Jewell W Comparison of immunohistochemistry by automated cellular imaging system (ACIS) versus fluorescence in-situ hybridization in the evaluation of HER-2/neu expression in primary breast carcinoma Histopathology 2006 48 3 258 267 10.1111/j.1365-2559.2005.02322.x 16430472

[B31] Ellis CM Dyson MJ Stephenson TJ Maltby EL HER2 amplification status in breast cancer: a comparison between immunohistochemical staining and fluorescence in situ hybridisation using manual and automated quantitative image analysis scoring techniques J Clin Pathol 2005 58 7 710 714 15976337 10.1136/jcp.2004.023424 PMC1770709

[B32] Hatanaka Y Hashizume K Kamihara Y Itoh H Tsuda H Osamura RY Tani Y Quantitative immunohistochemical evaluation of HER2/neu expression with HercepTestTM in breast carcinoma by image analysis Pathol Int 2001 51 1 33 36 10.1046/j.1440-1827.2001.01162.x 11148461

[B33] Joshi AS Sharangpani GM Porter K Keyhani S Morrison C Basu AS Gholap GA Gholap AS Barsky SH Semi-automated imaging system to quantitate Her-2/neu membrane receptor immunoreactivity in human breast cancer Cytometry A 2007 71 5 273 285 17323351 10.1002/cyto.a.20374

[B34] Skaland I Ovestad I Janssen EA Klos J Kjellevold KH Helliesen T Baak JP Comparing subjective and digital image analysis HER2/neu expression scores with conventional and modified FISH scores in breast cancer J Clin Pathol 2008 61 1 68 71 10.1136/jcp.2007.046763 17412872

[B35] Kononen J Bubendorf L Kallioniemi A Barlund M Schraml P Leighton S Torhorst J Mihatsch MJ Sauter G Kallioniemi OP Tissue microarrays for high-throughput molecular profiling of tumor specimens Nat Med 1998 4 7 844 847 10.1038/nm0798-844 9662379

[B36] Makretsov N Gilks CB Coldman AJ Hayes M Huntsman D Tissue microarray analysis of neuroendocrine differentiation and its prognostic significance in breast cancer Hum Pathol 2003 34 10 1001 1008 10.1053/S0046-8177(03)00411-8 14608533

[B37] Liu CL Prapong W Natkunam Y Alizadeh A Montgomery K Gilks CB Rijn M van de Software tools for high-throughput analysis and archiving of immunohistochemistry staining data obtained with tissue microarrays Am J Pathol 2002 161 5 1557 1565 12414504 10.1016/S0002-9440(10)64434-3 PMC1850765

[B38] Liu CL Montgomery KD Natkunam Y West RB Nielsen TO Cheang MC Turbin DA Marinelli RJ Rijn M van de Higgins JP TMA-Combiner, a simple software tool to permit analysis of replicate cores on tissue microarrays Mod Pathol 2005 18 12 1641 1648 16258508 10.1038/modpathol.3800491

[B39] The R Project for Statistical Computing http://www.r-project.org

[B40] GPEC TMA Viewer http://www.gpecimage.ubc.ca

[B41] Jensen KC Turbin DA Leung S Miller MA Johnson K Norris B Hastie T McKinney S Nielsen TO Huntsman DG New cutpoints to identify increased HER2 copy number: analysis of a large, population-based cohort with long-term follow-up Breast Cancer Res Treat 2008 112 3 453 9 10.1007/s10549-007-9887-y 18193353

[B42] Efron BTR An Introduction to the Bootstrap 1993 Boca Raton: CHAPMAN & HALL/CRC

[B43] Lehr HA Jacobs TW Yaziji H Schnitt SJ Gown AM Quantitative evaluation of HER-2/neu status in breast cancer by fluorescence in situ hybridization and by immunohistochemistry with image analysis Am J Clin Pathol 2001 115 6 814 822 10.1309/AJ84-50AK-1X1B-1Q4C 11392876

[B44] Thor A HER2–a discussion of testing approaches in the USA Ann Oncol 2001 12 Suppl 1 S101 107 10.1023/A:1011120309910 11521714

[B45] Gown AM Current issues in ER and HER2 testing by IHC in breast cancer Mod Pathol 2008 21 Suppl 2 S8 S15 10.1038/modpathol.2008.34 18437174

[B46] Moeder CB Giltnane JM Harigopal M Molinaro A Robinson A Gelmon K Huntsman D Camp RL Rimm DL Quantitative justification of the change from 10% to 30% for human epidermal growth factor receptor 2 scoring in the American Society of Clinical Oncology/College of American Pathologists guidelines: tumor heterogeneity in breast cancer and its implications for tissue microarray based assessment of outcome J Clin Oncol 2007 25 34 5418 5425 10.1200/JCO.2007.12.8033 18048824

[B47] Rhodes A Borthwick D Sykes R Al-Sam S Paradiso A The use of cell line standards to reduce HER-2/neu assay variation in multiple European cancer centers and the potential of automated image analysis to provide for more accurate cut points for predicting clinical response to trastuzumab Am J Clin Pathol 2004 122 1 51 60 10.1309/E9B55JYHD84L8Y17 15272530

[B48] Thomson TA Hayes MM Spinelli JJ Hilland E Sawrenko C Phillips D Dupuis B Parker RL HER-2/neu in breast cancer: interobserver variability and performance of immunohistochemistry with 4 antibodies compared with fluorescent in situ hybridization Mod Pathol 2001 14 11 1079 1086 10.1038/modpathol.3880440 11706067

[B49] Santinelli A Baccarini M Colanzi P Stramazzotti D Fabris G Immunohistochemical evaluation of HER-2/neu expression in infiltrating breast carcinoma: a study of reproducibility Anal Quant Cytol Histol 2002 24 1 54 62 11865950

[B50] Bloom K Harrington D Enhanced accuracy and reliability of HER-2/neu immunohistochemical scoring using digital microscopy Am J Clin Pathol 2004 121 5 620 630 10.1309/Y73U8X72B68TMGH5 15151201

[B51] Hashizume K Hatanaka Y Kamihara Y Kato T Hata S Akashi S Kato T Koyatsu J Tani Y Tsujimoto M Interlaboratory comparison in HercepTest assessment of HER2 protein status in invasive breast carcinoma fixed with various formalin-based fixatives Appl Immunohistochem Mol Morphol 2003 11 4 339 344 14663361 10.1097/00129039-200312000-00011

[B52] Gouvea AP Milanezi F Olson SJ Leitao D Schmitt FC Gobbi H Selecting antibodies to detect HER2 overexpression by immunohistochemistry in invasive mammary carcinomas Appl Immunohistochem Mol Morphol 2006 14 1 103 108 10.1097/01.pai.0000155794.64525.11 16540740

[B53] Ainsworth R Bartlett JM Going JJ Mallon EA Forsyth A Richmond J Angerson W Watters A Dunne B IHC for Her2 with CBE356 antibody is a more accurate predictor of Her2 gene amplification by FISH than HercepTest in breast carcinoma J Clin Pathol 2005 58 10 1086 1090 16189156 10.1136/jcp.2004.021576 PMC1770743

[B54] Lopez-Guerrero JA Navarro S Noguera R Almenar S Pellin A Vazquez C Llombart-Bosch A Histological tumor grade correlates with HER2/c-erB-2 status in invasive breast cancer: a comparative analysis between immunohistochemical (CB11 clone and Herceptest), FISH and differential PCR procedures Arkh Patol 2003 65 1 50 55 12669615

[B55] Tsuda H Tani Y Hasegawa T Fukutomi T Concordance in judgments among c-erbB-2 (HER2/neu) overexpression detected by two immunohistochemical tests and gene amplification detected by Southern blot hybridization in breast carcinoma Pathol Int 2001 51 1 26 32 10.1046/j.1440-1827.2001.01163.x 11148460

[B56] Tsuda H Sasano H Akiyama F Kurosumi M Hasegawa T Osamura RY Sakamoto G Evaluation of interobserver agreement in scoring immunohistochemical results of HER-2/neu (c-erbB-2) expression detected by HercepTest, Nichirei polyclonal antibody, CB11 and TAB250 in breast carcinoma Pathol Int 2002 52 2 126 134 10.1046/j.1440-1827.2002.01327.x 11940217

[B57] Sapino A Coccorullo Z Cassoni P Ghisolfi G Gugliotta P Bongiovanni M Arisio R Crafa P Bussolati G Which breast carcinomas need HER-2/neu gene study after immunohistochemical analysis? Results of combined use of antibodies against different c-erbB2 protein domains Histopathology 2003 43 4 354 362 10.1046/j.1365-2559.2003.01708.x 14511254

[B58] Vincent-Salomon A MacGrogan G Couturier J Arnould L Denoux Y Fiche M Jacquemier J Mathieu MC Penault-Llorca F Rigaud C Calibration of immunohistochemistry for assessment of HER2 in breast cancer: results of the French multicentre GEFPICS study Histopathology 2003 42 4 337 347 10.1046/j.1365-2559.2003.01598.x 12653945

[B59] Roche PC Suman VJ Jenkins RB Davidson NE Martino S Kaufman PA Addo FK Murphy B Ingle JN Perez EA Concordance between local and central laboratory HER2 testing in the breast intergroup trial N9831 J Natl Cancer Inst 2002 94 11 855 857 12048274 10.1093/jnci/94.11.855

[B60] Ellsworth RE Ellsworth DL Patney HL Deyarmin B Love B Hooke JA Shriver CD Amplification of HER2 is a marker for global genomic instability BMC Cancer 2008 8 297 18854030 10.1186/1471-2407-8-297 PMC2571108

[B61] Bishop JW Marcelpoil R Schmid J Machine scoring of Her2/neu immunohistochemical stains Anal Quant Cytol Histol 2002 24 5 257 262 12408558

[B62] Wen C-H Lee J-J Design and production of color calibration targets for digital input devices Proceedings of the Photonics Taiwan 2000: 26 July 2000; Taipei, Taiwan 2000 148

[B63] Walker RA Quantification of immunohistochemistry–issues concerning methods, utility and semiquantitative assessment I Histopathology 2006 49 4 406 410 10.1111/j.1365-2559.2006.02514.x 16978204

[B64] Taylor CR Levenson RM Quantification of immunohistochemistry–issues concerning methods, utility and semiquantitative assessment II Histopathology 2006 49 4 411 424 10.1111/j.1365-2559.2006.02513.x 16978205

